# GeneSurrounder: network-based identification of disease genes in expression data

**DOI:** 10.1186/s12859-019-2829-y

**Published:** 2019-05-06

**Authors:** Sahil D. Shah, Rosemary Braun

**Affiliations:** 1 Engineering Sciences and Applied Mathematics, Northwestern University, Evanston, USA; 2 Biostatistics, Feinberg School of Medicine, Chicago, USA; 3 Northwestern Institute on Complex Systems, Northwestern University, Evanston, USA

**Keywords:** Networks, Pathways, Gene expression, Systems biology, Algorithms

## Abstract

**Background:**

A key challenge of identifying disease–associated genes is analyzing transcriptomic data in the context of regulatory networks that control cellular processes in order to capture multi-gene interactions and yield mechanistically interpretable results. One existing category of analysis techniques identifies groups of related genes using interaction networks, but these gene sets often comprise tens or hundreds of genes, making experimental follow-up challenging. A more recent category of methods identifies precise gene targets while incorporating systems-level information, but these techniques do not determine whether a gene is a driving source of changes in its network, an important characteristic when looking for potential drug targets.

**Results:**

We introduce GeneSurrounder, an analysis method that integrates expression data and network information in a novel procedure to detect genes that are sources of dysregulation on the network. The key idea of our method is to score genes based on the evidence that they influence the dysregulation of their neighbors on the network in a manner that impacts cell function. Applying GeneSurrounder to real expression data, we show that our method is able to identify biologically relevant genes, integrate pathway and expression data, and yield more reproducible results across multiple studies of the same phenotype than competing methods.

**Conclusions:**

Together these findings suggest that GeneSurrounder provides a new avenue for identifying individual genes that can be targeted therapeutically. The key innovation of GeneSurrounder is the combination of pathway network information with gene expression data to determine the degree to which a gene is a source of dysregulation on the network. By prioritizing genes in this way, our method provides insights into disease mechanisms and suggests diagnostic and therapeutic targets. Our method can be used to help biologists select among tens or hundreds of genes for further validation. The implementation in R is available at github.com/sahildshah1/gene-surrounder.

**Electronic supplementary material:**

The online version of this article (10.1186/s12859-019-2829-y) contains supplementary material, which is available to authorized users.

## Background

The advent of high–throughput transcription profiling technologies has enabled identification of genes and pathways associated with disease, providing new avenues for precision medicine. A key challenge is to analyze this data in the context of the regulatory networks that control cellular processes, while still obtaining insights that can be used to design new diagnostic and therapeutic interventions. It is thus necessary to develop methods that analyze omic data in the context of the full network of interactions, while still providing specific, targetable gene-level findings.

The most common method for detecting gene-association is via differential expression analysis, in which each gene is independently tested for significant differences in mean expression between phenotypes [1]. However, while differential expression analysis can identify specific (and hence targetable) disease-associated genes, it does not take into consideration the network of molecular interactions that govern cellular function, limiting the mechanistic insights that can be derived from the data. As a result, this analysis can miss crucial multi-gene interactions that underlie complex phenotypes. Since biological systems are complex and expression data is typically noisy, the multi-gene mechanisms that underlie a disease may be detectable across multiple studies, but the individual genes contributing to those mechanisms may vary from one study to the next. As a result, differential expression analysis can exhibit poor agreement between different studies of the same conditions [2–4].

Maps of experimentally derived molecular interaction networks contained in pathway databases and the growth of analysis techniques that infer context-specific interaction networks have enabled the development of methods that integrate systems level information with expression data. KEGG [5], for example, is a well-established pathway database that organizes genes into hundreds of individual networks corresponding to biological processes. One use of interaction networks has been to identify *groups* of related genes underlying a biological mechanism. By incorporating systems-level information, these pathway analysis techniques can capture multi-gene interactions, yielding mechanistically interpretable results that are more reliable than single–gene analyses [2–4]. Pathway analysis techniques can be broadly grouped into three categories: ‘functional scoring methods’, ‘topology methods’, and ‘active modules tools.’ Functional scoring methods, such as GSEA [6], identify groups of genes that are enriched for association with the phenotype of interest. Topology methods, such as SPIA [7] and CePa [8, 9], also identify groups of genes that are enriched for association, but augment functional scoring methods with additional information about the network of interactions between the genes. Active modules tools, such as jActiveModules [10], HotNet [11], and COSINE [12], attempt to find disease associated subnetworks within pathways. These methods integrate systems-level information with expression data to identify groups of related genes.

While pathway analysis techniques integrate systems–level information with omic data to provide functional interpretations of the dataset, the “significant pathways” identified by such analyses often comprise tens or hundreds of genes, making experimental follow-up challenging. Additionally, boundaries between pathways are often arbitrary, thus potentially neglecting key interactions. Moreover, many techniques rely on user–settable parameters and ad-hoc heuristics that depend on network size, limiting their interpretability and reliability [4, 13]. Together, these issues point to the need for analysis techniques that integrate network and omics data to identify *precise* gene targets for follow-up studies.

Early efforts to identify precise gene targets while incorporating systems-level information include ENDEAVOUR [14] and GeneWanderer [15]. ENDEAVOUR takes in as input various data sources (such as literature abstracts and protein-protein interactions) and prioritizes genes based on their similarity to genes known to be involved in the disease. GeneWanderer uses protein-protein interaction networks and identifies gene targets based on distance to known disease genes on the network. However, these methods require knowledge of mechanisms known to be associated with the disease. Later analysis techniques – such as a method that uses the Laplacian kernel [16], an extension of SPIA [17], and nDGE [18] – addressed this issue and do not require knowledge of disease associated mechanisms to identify precise gene targets. The first method uses a protein association network, recomputes distances using the Laplacian kernel, and finds disease genes based on “neighboring” differential expression. Since the distances are recomputed, the neighbors could include genes that are not neighbors on the original network. In other words, this method uses indirect interactions instead of direct interactions, complicating the interpretation. In the extension of SPIA [17], disease genes are found by propagating changes in expression along the edges of the individual pathway so that each gene is scored for disease-association according to its own change in expression combined with the change in expression of its upstream neighbors. Since each pathway is considered separately and the pathways have artificial (sometimes overlapping) boundaries, an individual pathway could exclude genes that are on a global network (i.e. union of the individual pathways). nDGE takes in as input expression profiles and for a putative disease gene class conditionally identifies its co-regulated and actively co-regulated neighbors. While powerful, each of these is limited in its treatment of the networks. These methods either do not consider direct interactions between genes on a global network ([16] uses indirect interactions based on the Laplacian kernel and [17] considers each KEGG pathway separately) or infer interactions based on correlations (e.g., [18]). Thus due to the limitations of the previously described techniques, an analysis technique that takes into account direct interactions between genes globally may prove useful in identifying targets and the effect they have on the network.

Most recently, LEAN [19] was developed to use direct interactions on a global interaction network and find disease genes by scoring the differential expression of “local subnetworks.” LEAN scores each gene for disease-association according to the enrichment of its immediate neighbors. Thus, LEAN’s algorithm restricts its focus to a local subnetwork that only considers nearest neighbors. As a result, LEAN only identifies genes based on the changes in expression of a given gene’s local subnetwork, but can not determine whether that gene is actually the source of changes in its neighborhood or on the network; an important characteristic when looking for potential targetable disease genes for use in precision medicine.

The goal of the present work is to combine pathway network information with gene expression data to determine the degree to which a gene is a source of dysregulation on the network. We present a novel analysis technique, GeneSurrounder, that takes into account the complex structure of interaction networks to identify specific disease-associated genes from expression data. The key idea of our method is to score genes based on the evidence that they influence the dysregulation of their neighbors on the network in a manner that impacts cell function. In this way, the genes returned by our method may be considered sources of “disruption” on the network and therefore candidate targets for therapeutics. We thus seek to identify genes with two defining characteristics: (*i*) they appear to influence other genes nearby in the network, as evidenced by strongly correlated expression with nearby genes; and (*ii*) their dysregulation is associated with disease, as evidenced by a pattern of (progressively weaker) differential expression centered about that gene. By finding these genes, our method identifies candidate genes that are “disruptive” to the mechanisms underlying a given phenotype and does so without any reliance on user-set parameters or arbitrary pathway boundaries.

In this manuscript, we describe the GeneSurrounder algorithm and apply it to data from three independent studies of ovarian cancer to demonstrate its use, evaluate the reproducibility of its results, and demonstrate the methodological and biological validity of our approach. In order to evaluate the algorithm, we evaluate its cross-study concordance, i.e., its consistency across different data sets measuring the same phenotype. We compare the cross-study concordance of GeneSurrounder’s results to that of standard differential expression analysis, and find that genes identified as sources of pathway disruption by GeneSurrounder are more consistently identified across the various studies than are differentially expressed genes. We also compare our method to LEAN, and show that genes identified by GeneSurrounder are more consistent across studies than both LEAN and differential expression analysis. We demonstrate that our method represents an integration of pathway and expression data to yield results that are not solely driven by either alone and find that it identifies genes associated with ovarian cancer. Together, these results suggest that GeneSurrounder reproducibly detects functionally-relevant genes by integrating gene expression and network data. Our novel analysis approach complements existing gene– and pathway–based analysis strategies to identify specific genes that control disease–associated pathways, providing a new strategy for identifying promising therapeutic targets.

## Methods

Our goal is to identify candidate disease genes by analyzing gene expression data in the context of interaction networks to discover genes that drive the behavior of pathways associated with disease. We thus seek to identify genes with two defining characteristics: (*i*) they appear to influence other genes nearby in the network, as evidenced by strongly-correlated expression with nearby genes; and (*ii*) their dysregulation is associated with disease, as evidenced by a pattern of differential expression centered about that gene. Since the ‘extent’ of dysregulation of a given gene on a global gene network is not known a priori, we score the gene separately for every neighborhood size on the network (i.e. genes one ‘hop’ away, genes up to two ‘hops’ away, etc) and then return the results for the highest scoring neighborhood. Genes with significantly high-scoring neighborhoods may then be prioritized for follow-up experiments.

**Fig. 1:**
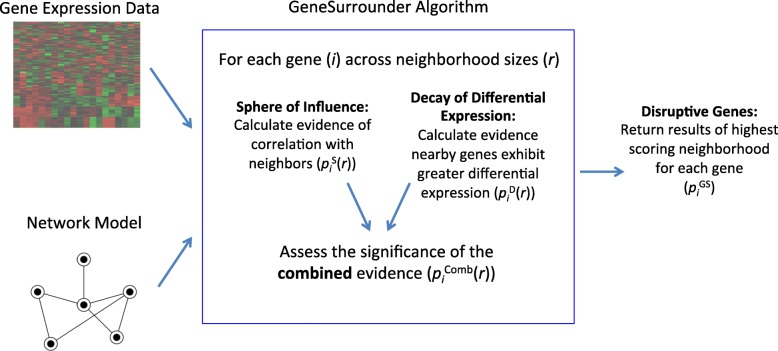
Overview of GeneSurrounder algorithm. The algorithm incorporates systems–level information, in the form of a network model of cellular interactions, with gene expression data to identify the genes that control disease–associated mechanisms. The algorithm than identifies “disruptive” genes by assessing the significance of the combined evidence that (1) a gene has a influence on others in the network and (2) that its influence is driving disease

To this end, the GeneSurrounder method consists of two tests that are run independently of each other (Fig. 1) and then combined, for every neighborhood size on the network. To determine if the putative disease gene is a “disruptive” candidate disease gene meeting both criteria, the results for the highest scoring neighborhood are returned. To prioritize genes, our method is applied exhaustively to each assayed gene in a transcriptomic data set, and the results from each gene’s highest scoring neighborhood are compared to rank the genes.

The algorithm takes as input gene expression data and a network model of cellular interactions derived from a pathway database. In order to consider the full scope of a gene’s interactions and avoid artificially imposed pathway boundaries, we create a global KEGG network by merging the individual pathways so that the links which are in at least one KEGG pathway will be part of the new global network (i.e., the graph union of all pathways). We then consider the largest connected component of the resulting network in our algorithm. Using this global network and gene expression data, we compute evidence for each of the above criteria as follows.

### Does a gene appear to influence its neighbors in the network? Evidence of “Sphere of Influence”

**Fig. 2:**
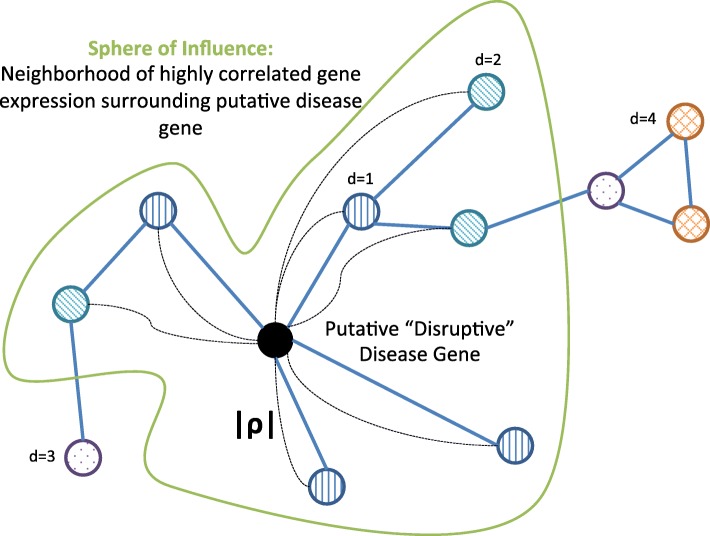
Procedure for Sphere of Influence. The Sphere of Influence computation tests if a putative driver gene is more correlated with its neighbors than a random sample of genes

If a gene is a source of regulatory control or disruption, we may expect to see that its behavior is correlated with that of its neighbors. The first step, dubbed “Sphere of Influence,” assesses if a candidate gene *i* meets this criterion by testing if gene *i* is more strongly correlated with its network neighbors than would be expected by chance (Fig. 2), compared to a random sample of genes. The first step, therefore, of the Sphere of Influence procedure is to calculate the Spearman rank correlation *ρ*_*ij*_ between gene *i* and every other gene *j* assayed and on the network. From this set of correlations, we calculate the observed total (absolute) correlation between gene *i* and its neighbors within a neighborhood of radius *r*,

1 $$ C_{i}(r) = \sum_{\{j:d_{ij}{\leq}r\}} |\rho_{ij}| \,,   $$

where *d*_*ij*_ indicates the geodesic distance of gene *j* from gene *i* on the network.

In order to compute the distribution of total correlation under the null hypothesis that it is drawn from a random sample of genes, we re-sample with replacement from the set of correlations between gene *i* and every other gene *j* and recompute Eq. 1. This procedure effectively redistributes the gene–gene correlations about the network, enabling a comparison of gene *i*’s influence in the true network neighborhood to its influence on a random selection of genes. This step tests the so–called “competitive null” described in [3]; that is, whether gene *i* has a greater correlation with genes in its neighborhood than would be a expected from a random set of genes.

The null distribution of the total absolute correlation for gene *i* as a function of the neighborhood radius is computed using 10^3^ re-samplings, and the observed total absolute correlation is compared to the re-sampled null distribution, yielding a “Sphere of Influence” *p*–value at each neighborhood radius for gene *i*, $p^{\mathrm {S}}_{i}(r)$, that quantifies whether *i* is more correlated with its neighbors than expected by chance, evidence that it may be an influential gene.

### Does the gene’s neighborhood exhibit an association with phenotype? Evidence of “Decay of Differential Expression”

The previous step tests whether gene *i* is strongly correlated with its network neighbors, independent of phenotype. If a gene is a source of disease-associated disruption, we may expect that it and its neighbors will exhibit differential expression. We thus now turn our attention to whether the gene and its neighbors also exhibit an association with the phenotype of interest. In particular, if a gene *i* is a source of dysregulation that drives the phenotype, we would expect that gene *i* and its close neighbors will be differentially expressed, while genes farther away in the network will exhibit weaker differential expression. In other words, we expect a pattern of differential expression that is strongly localized about *i* and decays as one moves farther from it in the network.

**Fig. 3:**
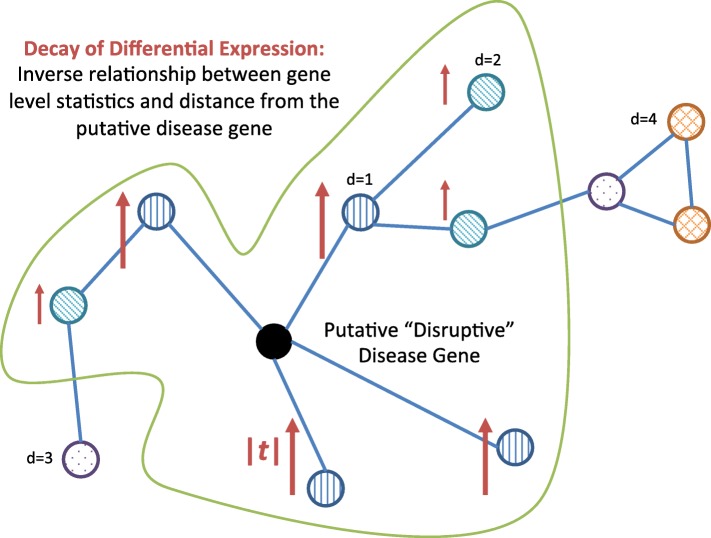
Procedure for Decay of Differential Expression. The Decay of Differential Expression computation tests if the discordance between differential expression and distance from the driver gene is greater with the phenotype labels we observe than with

Hence, the second calculation, “Decay of Differential Expression,” tests whether the magnitude of differential expression of other genes *j* in the neighborhood is inversely related to the distance *d*_*ij*_ of gene *j* from gene *i* (Fig. 3).

In order to do this, we must first compute a gene–level statistic *g*_*j*_ that quantifies the magnitude of *j*’s association with the outcome of interest. We then quantify the “decay of differential expression” with the Kendall *τ*_*B*_ rank correlation coefficient between the differential expression and distance from gene *i*.

The observed discordance is

2 $$ D_{i}(r) = \tau_{B}\left(\{g_{j}:d_{ij}{\leq}r\}, \{d_{ij}:d_{ij}{\leq}r\}\right)\,,   $$

where *d*_*ij*_ is the geodesic distance between gene *j* and gene *i*.

To assess the statistical significance of *D*_*i*_(*r*), we randomly permute the phenotype labels and recompute the gene–level association statistics *g*_*j*_ under the null hypothesis that the genes are not meaningfully associated with the phenotype. We then use the permuted $g^{*}_{j}$ to recompute $D^{*}_{i}$ according to Eq. 2. A set of 10^3^ such re-computations forms a reference distribution against which we compare the observed *D*_*i*_ to obtain a *p* value $p^{\mathrm {D}}_{i}(r)$ as the fraction of $D^{*}_{i}<D_{i}$.

It should be noted here that while $p^{\mathrm {S}}_{i}(r)$ (above) was obtained by randomly permuting *genes*, $p^{\mathrm {D}}_{i}(r)$ is obtained by permuting the class labels. An important feature of the latter is that it preserves correlations between genes that were found in the $p^{\mathrm {S}}_{i}(r)$ calculation. In consequence, the null models, and hence the interpretations, of the two tests differ. $p^{\mathrm {S}}_{i}(r)$ quantifies whether the neighborhood surrounding gene *i* is more strongly correlated with it than a random set of genes would be (independent of phenotype), testing the so–called “competitive null” [3]. In contrast, $p^{\mathrm {D}}_{i}(r)$ assesses whether the neighborhood surrounding gene *i* is more strongly associated with the phenotype of interest than those same genes would be with randomly–assigned phenotype labels (preserving the organization of genes in the network), thus testing the so–called “self-contained null” [3]. That is, it tests whether a specific set of genes in a neighborhood is more strongly associated with the phenotype of interest than the same set of genes would be for a random phenotype.

Because these two procedures permute orthogonal axes (genes *vs.* samples), they provide two independent tests with independent interpretations: $p^{\mathrm {S}}_{i}(r)$ tests whether gene *i* influences its neighbors, and $p^{\mathrm {D}}_{i}(r)$ tests whether that neighborhood is associated with disease. We then combine these independent pieces of evidence into a single assessment, as described below.

### Combined evidence

At this point in our algorithm, the Sphere of Influence and Decay of Differential Expression procedures have been run independently of each other, but neither component is sufficient by itself to determine if putative disease gene *i* is in fact a “disruptive” candidate disease gene meeting both criteria. Therefore, the last step our method performs is to combine the *p*-values outputted by each component $\left (p^{\mathrm {S}}_{i}(r)\ \text {and}\ p^{\mathrm {D}}_{i}(r)\right)$ using Fisher’s method [20],

3 $$ X^{2} = -2\left(\ln\left(p^{\mathrm{S}}_{i}(r)\right)+\ln\left(p^{\mathrm{D}}_{i}(r)\right)\right)\,.   $$

*X*^2^ follows a *χ*^2^ distribution with 4 degrees of freedom, which can be used compute $p^{\text {Comb}}_{i}(r)$, the combined evidence that gene *i* is a “disruptive” gene.

### Neighborhood size

Above we described the Sphere of Influence and Decay of Differential Expression procedures for a fixed radius (*r*), but different genes may have different extents of influence on the network, and this extent is not known a priori. Therefore, we have devised our analysis technique to apply the Sphere of Influence, Decay of Differential Expression, and Combined Evidence calculations to the neighborhood of every radius (up to *D* the diameter of the network). The *p*-value our method outputs for each gene $\left (p^{\text {GS}}_{i}\right)$, therefore, is the smallest $p^{\text {Comb}}_{i}(r)$ across all distances.

To evaluate the significance of $p^{\text {GS}}_{i}$, we then apply a Bonferroni correction to the significance threshold to conservatively adjust for the multiple hypothesis tests that we perform when applying our method to the neighborhoods of each radius. Since the number of neighborhoods (and therefore number of tests) is determined by the diameter of the network, we scale the significance threshold by the diameter of the network to determine whether a gene was significantly found to be “disruptive” in the data. Adjustment for the multiplicity of genes tested is achieved through permutation as previously described [6, 21, 22]; this has the important benefit of preserving the biologically-relevant dependency structure between genes [6, 22].

### Example of GeneSurrounder steps applied to an example gene

**Fig. 4:**
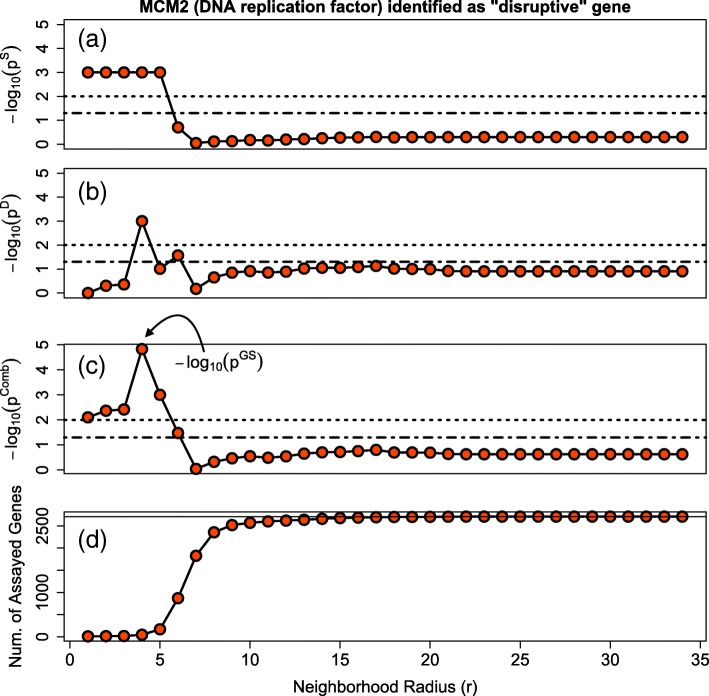
Illustration of Method. Displayed are the results for the gene MCM2 when our algorithm was applied to Ovarian Cancer Study GSE14764. **a** shows − log10(*p*_Sphere_) vs the Neighborhood Radius. **b** shows − log10(*p*_Decay_) vs the Neighborhood Radius. **c** shows − log10(*p*_Combined_) vs the Neighborhood Radius. **d** shows the Number of Assayed Genes vs the Neighborhood Radius. In the top three plots, the dashed and dotted lines correspond to a significance level of 0.05 and 0.01 respectively. In the bottom plot, the solid line corresponds to the total number of genes assayed and on the network

To illustrate the components of the GeneSurrounder computation, we present the results for each component of our algorithm as applied to gene *MCM2* using data from one study of high-vs-low grade ovarian cancer [23] (GEO accession GSE14764). In Fig. 4, each of the first three plots (from top to bottom) displays the − log10(*p*) from the Sphere of Influence, Decay of Differential Expression and Combined components of our method. Since we compute these values as a function of network neighborhood size surrounding that gene, the *p*-values are plotted against the neighborhood radius (i.e. radius of geodesic distance from the putative “disruptive” disease gene *MCM2*.)

Figure 4a (Sphere of Influence) illustrates the dilution of influence with distance and the effect that the size (i.e. number of assayed genes) of a neighborhood has on the decrease of influence. The putative disease gene in this example, *MCM2*, has significant influence in neighborhoods near to it, but this influence falls off and stays non-significant at far-away distances. The largest difference occurs between a radius of 5 and 6, where the number of assayed genes within the neighborhood (Fig. 4d) increases sharply, contributing to the dilution of *MCM2*’s influence.

Figure 4b (Decay of Differential Expression) indicates a significant concentration of differential expression for neighborhoods with radii of 4–6. We observe that small neighborhoods immediately near a putative disease gene are not big enough to detect a decaying pattern of differential expression, such that the localized differential expression is only detectable at with a radius of at least 4. At the other end, big neighborhoods are too diverse to exhibit a consistent decay of differential expression; like the sphere of influence, the significance of the decay of differential expression flattens out at large distances.

Figure 4c illustrates the results of combining the results for each neighborhood. The *p*-value our method outputs for each gene is the most significant $p^{\text {Comb}}_{i}(r)$ across all neighborhood radii; for *MCM2* in this study, this occurs at a neighborhood radius of 4 with *p*_GS_=1.48e−05. Since our method returns the smallest $p^{\text {Comb}}_{i}(r)$ for each gene (equivalently, the largest $-\log _{10} p^{\text {Comb}}_{i}(r)$) and the smallest $p^{\text {Comb}}_{i}(r)$ of *MCM2* is highly significant, *MCM2* would be identified as a central candidate disease gene of high grade ovarian cancer. From a biological standpoint, this finding is sensible: *MCM2* is a DNA replication factor, and therefore likely plays a role in the aggressive proliferation associated with high-grade ovarian carcinoma.

## Results

### Application to ovarian cancer data with global KEGG network model

We apply our algorithm to three gene expression data sets of high-vs-low grade ovarian cancer from the publicly available and curated collection ‘curatedOvarianData’ [23] to illustrate the components of the GeneSurrounder method and evaluate its performance. In order to test our algorithm, we evaluate its cross-study concordance, i.e., its consistency across different data sets that are measuring the same conditions, as previously described [4]. The intuition underlying this approach is that methods that detect true biological signals should find them across different data sets measuring the same conditions. To test this we use data from three independent studies of gene expression in high and low grade ovarian cancer tumors (Table 1). The data were obtained from the Bioconductor package ‘curatedOvarianData’ [23], a project designed to facilitate meta-analysis by providing data that has been harmonized to ensure that clinical measurements (such as grade) are comparable across studies. Gene expression data was preprocessed by the original authors using established normalization techniques, and no further preprocessing was required. Following our previous work [4], we confine our analysis to genes assayed in common across all datasets and which appear in the KEGG network; a total of 2709 genes meet these criteria.

**Table 1:** Ovarian cancer datasets used in this study

GEO Accession No.	*N*(low-grade)	*N*(high-grade)
GSE14764	24	44
GSE17260	67	43
GSE9891	103	154

Comparisons were made between low– and high–grade serous ovarian carcinoma using public data. Sample sizes for each group in each dataset are given. The data are publicly accessible and available as part of the curatedOvarianData package [23]

Our method combines two independent sources of information — the gene expression data and a pathway network model — to detect the disruptive genes of the phenotype under consideration. We use the same global network model for each study, which we have constructed from KEGG pathways [5]. The KEGG database organizes experimentally derived pathway information into individual networks of functionally related molecules. In the KEGG representation, the nodes (i.e. vertices) are genes or gene products, and the links (i.e. edges) are cellular interactions. We create a global KEGG network to avoid the artificial boundaries between individual pathways by taking the graph union of the individual pathways, i.e. merging the pathways so that the links which are in at least one KEGG pathway will be part of the new global network. We then consider the largest connected component of the resulting network in our algorithm. This global network has *N*=4867 nodes, *L*=42874 edges, and a diameter *D*=34. Of the *N*=4867 nodes, 2709 of them are also amongst the 7680 genes assayed in all three ovarian cancer studies.

We apply our method to each of the ovarian cancer studies with the global gene network to calculate the combined evidence $p^{\text {Comb}}_{i}(r)$ for each of the 2709 genes *i* that are assayed and on the network. A table of the full results is provided as an Additional files 1, 2 and 3. With the results from each of the three ovarian cancer data sets, we evaluate not only the cross-study concordance of our analysis technique, but also its ability to identify biologically relevant genes and truly integrate pathway and expression data.

### Disruptive genes found by GeneSurrounder are associated with ovarian cancer

To evaluate GeneSurrounder’s ability to identify biologically relevant genes, we compare our results in all three ovarian cancer studies (Table 2) to existing biological knowledge. Applying GeneSurrounder to the 2709 common genes between studies that were assayed and on the network, we generated three distinct ranked lists of genes for each study based on the computed $p^{\text {GS}}_{i}$ value. To compare these results to existing biological knowledge, we consider genes that pass our Bonferroni corrected threshold (at significance level *α*=0.05 and with a diameter of *D*=34, our Bonferroni corrected threshold is − log10(*p*)≥2.83) in all three studies (Table 2).

**Table 2:** “Disruptive” disease genes in high-grade ovarian cancer consistently found by GeneSurrounder

	−log10*p*^GS^		
Gene	GSE14764	GSE17260	GSE9891
ADRB3	3.033	2.933	3.554
AURKA	2.865	3.383	3.716
CDC45	4.270	3.741	4.830
CDC7	4.386	3.769	4.830
DBF4	4.270	3.769	4.830
IL7	3.055	2.898	2.910
ITGAM	2.961	3.024	3.094
MCM2	4.830	3.372	4.830
MCM3	4.830	3.383	4.830
MCM4	4.830	3.394	4.830
MCM5	4.830	3.372	4.830
MCM6	4.830	3.428	4.830
ORC4	4.386	3.172	4.830
ORC6	4.386	3.691	4.830
TTK	2.904	3.089	4.830

At a threshold of *p*=0.05 and with a diameter of *D*=34, the Bonferroni corrected threshold is −log10(*p*)≥2.83. Listed are the genes that pass this threshold in all three studies

We used the DOSE R package [24] to analyze the enrichment of these genes with Disease Ontology (DO) terms [25]. We found that the genes that pass our Bonferroni corrected threshold in at least one ovarian cancer study were significantly enriched with the DO term “ovarian cancer” (DOID:2394) (*p*=0.0000177). Furthermore, amongst these genes are three families of protein coding genes, *CDC* (involved in the cell division cycle), *MCM*, and *ORC* (both involved in DNA replication), with biological functions that support their role in ovarian cancer.

To further compare our results to existing biological knowledge, we found evidence in the literature that *CDC7*, *ORC6L*, and *DBF4* are associated specifically with ovarian cancer [26–28]. The inclusion of *CDC45* suggests the possibility that it is also associated with ovarian cancer. *CDC7* encodes for a cell division cycle protein and has been found to both predict survival and be a powerful anticancer target in ovarian cancer [26]. *ORC6L* encodes for a origin recognition complex that is crucial for the initiation of DNA replication and has been found to highly expressed in ovarian cancer [27]. *DBF4* encodes for a protein that activates the kinase activity of *CDC7* and was found to be associated with ovarian cancer [28]. The finding of these genes from studies of high-vs-low grade ovarian cancers suggests the possibility that they are not only involved in ovarian cancer but, more specifically, drive high grade ovarian cancer. A table of the full results is provided as an Additional files 1, 2 and 3.

### GeneSurrounder results represent a true integration of pathway and expression data

The method that we have developed combines gene expression data with an independent network model. To investigate whether our results are driven solely by either the network or the expression data or represent a true integration of biological knowledge (the pathway networks) and experimental data, we consider the association between our results, the centrality, and the differential expression for each gene. If the results were driven solely by the network, the evidence a gene is a disruptive gene would correlate strongly with its centrality in the network. We therefore calculate the correlation between our results and two different measures of centrality. If the results were driven solely by the expression data, the evidence a gene is a disruptive gene would correlate strongly with its differential expression We therefore calculate the correlation between our results and the differential expression for each of the studies. The results are given in Table 3. We find that for each of the studies, the correlations are small (on the order of 0.01), confirming that GeneSurrounder is not driven solely by network features or the expression data, but rather represents a true integration of biological knowledge (the pathway networks) with experimental data.

**Table 3:** Correlation between GeneSurrounder results and network/gene statistics

Network/Gene statistic	GSE14764	GSE17260	GSE9891
Degree Cor.	0.044	0.070	0.038
Betweenness Cor.	0.047	0.059	0.030
*p*_DE_ Cor.	0.060	0.103	− 0.051

The three columns are the rank correlation between GeneSurrounder results (*p*^GS^) and network/gene statistics (Degree, Betweenness, and *p*^DE^) across all genes in each dataset. The Degree and Betweenness are two different network centrality measures. The Degree is the number of connections a node has and the Betweenness is the fraction of shortest paths that passes through the node. *p*^DE^ is the *p*-value obtained from a standard differential expression *t*-test

### GeneSurrounder findings are more concordant than differential expression analysis

The intuition underlying evaluating cross-study concordance is that methods that detect true biological signals should find them across different data sets measuring the same conditions. To investigate the cross-study concordance of our analysis technique (i.e. its consistency across different data sets measuring the same conditions), we consider each pair of the three studies and calculate the correlation between our results. As a point of reference, we also calculate the correlation between the gene level statistics obtained using the customary *t*-test for differential expression. The results are given in Table 4. As mentioned earlier, methods that do not take into account systems-level information tend to have poor agreement between studies because the individual genes contributing to disease-associated mechanisms can vary from one study to the next. Indeed, we find that the cross-study concordance of differential expression results is remarkably low (Table 4). By contrast our method is 3.51—8.55 times more consistent than differential expression analysis. This cross–study concordance suggests that our method reliably detects biological effects reproducibly across studies.

**Table 4:** Cross study concordance of GeneSurrounder results compared to differential expression analysis and LEAN

Ovarian cancer study pair	*p*^GS^ Cor.	*p*^DE^ Cor.	*p*^LEAN^ Cor.
GSE14764 - GSE17260	0.342	0.040	0.056
GSE14764 - GSE9891	0.436	0.056	0.130
GSE17260 - GSE9891	0.485	0.138	0.290

The columns *p*^GS^ Cor., *p*^DE^ Cor., and *p*^LEAN^ Cor. are the Spearman rank correlations respectively between the results obtained from GeneSurrounder, differential expression analysis, and LEAN for each study pair

### GeneSurrounder findings are more concordant than LEAN

We also compare GeneSurrounder to LEAN, a recent method that also attempts to integrate gene expression and network data to identify significant genes. In contrast to our method, LEAN considers only the immediate neighborhood (i.e. at a radius of one) and assesses the enrichment of significant genes. To compare the performance of our analysis technique to LEAN, we compare their respective cross-study concordances. To ensure comparability between our method and LEAN, we use the same network and expression data for inputs to LEAN that we used for GeneSurrounder. Again, we consider each pair of the three studies and calculate the correlation between our results and the correlation between results of LEAN [19] (which is available as an R package on CRAN). The results are given in Table 4. We found that while LEAN is more consistent than the differential expression analysis, GeneSurrounder is more consistent than LEAN. That is, the list of “disruptive” genes detected by GeneSurrounder are more reproducible across studies than both differentially expressed genes and the results from LEAN.

### Application to bladder cancer data with global KEGG network model

As a further demonstration of our method, we apply our algorithm to three bladder cancer gene expression data sets from the publicly available and curated collection ‘curatedBladderData’ [29] (Additional file 7: Table S1). Bladder tumor samples in each data set are classified as either superficial (no invasion of the main muscle layer) or invasive (tumor growth into the main muscle layer), and we compare samples between these two groups. As in our application to ovarian cancer, the bladder cancer data was downloaded using the Bioconductor package ‘curatedBladderData’ without further processing. The same global KEGG network we created to analyze the ovarian cancer data was used to analyze the bladder cancer data. In order to compare results obtained from each of the bladder cancer datasets and compute cross-study concordance, we restricted each analysis (GeneSurrounder, differential expression, and LEAN) to the set of 2205 genes that were assayed in all three studies and were in the KEGG network. These were then filtered further to exclude both genes and samples with > 25*%* missing data in any study. After mapping gene symbols in the three bladder data sets to KEGG identifiers and filtering out genes with missing data, 1757 genes remained in common to all three bladder cancer studies.

We apply GeneSurrounder to these 1757 genes to identify genes passing the Bonferroni corrected threshold (at a threshold of *p*=0.05 and with a diameter of *D*=34, our Bonferroni corrected threshold is − log10(*p*)≥2.83) A table of the full results is provided as an Additional files 4, 5 and 6). Several genes are identified as statistically significant in all three studies (Additional file 7: Table S2); their functional roles in cell migration and adhesion (a mechanism required for the progression of tumors from “superficial” to “invasive”) further supports the ability of GeneSurrounder to detect biologically relevant signals.

As with the ovarian cancer data, we also evaluate our method’s correlation with network features and its cross–study concordance. We confirm that GeneSurrounder is not driven solely by network features or the bladder cancer expression data, but represents an integration of both (Additional file 7: Table S3). We also confirm that GeneSurrounder yields more reproducible results than competing analyses (Additional file 7: Table S4). While concordance values for all analysis methods were generally lower in the bladder cancer studies than in the ovarian cancer studies, we nevertheless find that GeneSurrounder is still more concordant than both differential expression analysis and LEAN. A more detailed description of these results, including discussion of significant genes, is provided in Additional file 7.

## Discussion

In this manuscript, we have developed and presented a new analysis technique, GeneSurrounder, that integrates a network model with expression data to identify individual genes that can be targeted therapeutically. Our analysis technique identifies “disruptive” genes — genes that impact pathway networks in a disease associated manner. The algorithm consists of two tests that are run independently of each other and then combined. The first test, Sphere of Influence, calculates the evidence that a putative disease gene is correlated with its neighbors, and the second test, Decay of Differential Expression, calculates the evidence that the neighbors of a putative disease gene are differentially expressed (with the magnitude of differential expression decreasing with distance).

We applied our algorithm to three gene expression data sets of high-vs-low grade ovarian cancer [23] and combined each of them with the same global network model that we constructed from KEGG pathways. With the results from each of the three ovarian cancer data sets, we evaluated our analysis technique. By applying our method to three different data sets measuring the same conditions, we were able to show that it yields consistent (i.e. concordant) results across studies, suggesting its ability to detect biologically meaningful associations that are reproducible across studies. We also compare our results to existing biological knowledge and find that our method identifies biologically relevant genes. To show that our method truly integrates pathway and expression data, we compare the results from our method to the results from a single gene analysis and the centrality of the genes in the network. Our positive results along these three dimensions of our analysis technique suggest that our method is a promising new strategy for identifying the genes that control disease.

As discussed in the Introduction, pathway analysis techniques such as GSEA [6], jActiveModules [10], and COSINE [12] use interaction networks and expression data to find groups of related disease-associated genes. GeneSurrounder, to make experimental follow-up easier, identifies precise gene targets rather than groups of tens or hundreds of genes. Efforts to identify individual genes, as our method does, have either required prior biological knowledge (as in ENDEAVOUR [14] and GeneWanderer [15]) or have not used direct interactions on a global network (as in [16], an extension of SPIA [17], and nDGE [18]). Our analysis technique addresses these shortcomings by using the shortest direct distance on a global network and not requiring any prior biological knowledge. LEAN [19] considers interactions on a global interaction network and is closest to our method in this respect, but restricts its focus to nearest neighbors on the network and does not determine whether a putative disease gene is the source of change on the network.

## Conclusions

The key innovation of GeneSurrounder is the combination of pathway network information with gene expression data to determine the degree to which a gene is a source of dysregulation on the network. GeneSurrounder employs a novel strategy by finding genes that both appear to influence nearby genes and cause dysregulation associated with the disease. Because GeneSurrounder considers every neighborhood size around a putative gene, it is able to identify disease genes that may have broad effects on the regulatory network (beyond nearest neighbors). GeneSurrounder thus provides a new avenue for identifying disease-associated genes by detecting genes that appear to be sources of change and could therefore be promising therapeutic targets.

While our method performs well in practice, there are limitations that bear consideration. We note that the network model that we use, KEGG, is not phenotype-specific (as are most pathway databases) and we therefore have to assume that the network does not change between conditions. Additionally, because KEGG (and other pathway databases) may not be complete, genes that are not annotated in any pathway cannot be considered in a GeneSurrounder analysis. Furthermore, as implemented our algorithm calculates geodesic distances between genes without taking into account the direction or type of interactions. However, we note that our approach as presented here could easily be modified to take in as input other kinds of networks (including context-specific computationally derived networks) and/or considering edge directionality by changing the gene-gene distance matrix that the Sphere of Influence and Decay of Differential computations use.

GeneSurrounder can be potentially generalized to other types of data. For instance, one might envision applying it to other kinds of omic data. For example, GeneSurrounder could potentially be extended to use genomic sequence data to identify epistatic interactions, evidenced by gene neighborhoods that have a high level of correlations in their genetic variants. Our method could also possibly be generalized for time-series gene expression data by either changing the gene-level statistics used by the algorithm or applying it separately to time points.

GeneSurrounder thus provides means to prioritize genes that are sources of disruption for a disease in the context of gene regulatory networks. By prioritizing genes in this way, our method provides insights into disease mechanisms and suggests diagnostic and therapeutic targets. Our method can be used to help biologists select among tens or hundreds of genes for further validation. Furthermore, it can be generalized to other kinds of networks (including context-specific networks) and omic data. This approach can not only be used directly to prioritize promising targets, but also suggests new strategies for integrating systems level information with omic data to identify, validate, and target disease mechanisms. We have made the implementation of our method available to researchers on GitHub at http://github.com/sahildshah1/gene-surrounder with the aim of furthering our understanding of statistical techniques to identify disease-associated genes.

## Additional files

Additional file 1 Table of the full results of GeneSurrounder applied to GSE14764. (CSV 258 kb)

Additional file 2 Table of the full results of GeneSurrounder applied to GSE17260. (CSV 259 kb)

Additional file 3 Table of the full results of GeneSurrounder applied to GSE9891. (CSV 262 kb)

Additional file 4 Table of the full results of GeneSurrounder applied to GSE13507. (CSV 463 kb)

Additional file 5 Table of the full results of GeneSurrounder applied to GSE19915.GPL5186. (CSV 185 kb)

Additional file 6 Table of the full results of GeneSurrounder applied to GSE32894. (CSV 362 kb)

Additional file 7 More detail on results of applying Gene Surroundeer to the Bladder Cancer Data. (PDF 117 kb)
