# Supplementary material for: GeneSurrounder: network-based identification of disease genes in expression data
Source: BMC Bioinformatics. 2019 May 6;20:229. doi: 10.1186/s12859-019-2829-y (PMC6503437; doi:10.1186/s12859-019-2829-y)
Supplement: Supplementary file 7 — More detail on results of applying Gene Surroundeer to the Bladder Cancer Data. (PDF 117 kb) [file 12859_2019_2829_MOESM7_ESM.pdf]

Supplement

GeneSurrounder Analysis of curatedBladderData

In this supplement, we detail the results of applying GeneSurrounder to data from the curatedBladderData package. The analysis parallels that of the ovarian cancer data described in the main manuscript. We apply our algorithm to three gene expression data sets of superficial-vs-invasive bladder cancer from the publicly available and curated collection 'curatedBladderData' [1] (Table S1). The three gene expression data sets and the KEGG network model have 2205 genes in common. After mapping gene symbols in the three bladder data sets to KEGG identifiers and filtering out genes with missing values in 25% or more of the samples in any study, 1757 genes remained in common to all three bladder cancer studies.

Disruptive genes found by GeneSurrounder are associated with invasive bladder cancer

To evaluate GeneSurrounder's ability to identify biologically relevant genes, we compare our results in all three bladder cancer studies (Table S2) to existing biological knowledge. Applying GeneSurrounder to the 1757 common genes between studies that were assayed and on the network, we generated three distinct ranked lists of genes for each study based on the computed p_i^{GS} value. To compare these results to existing biological knowledge, we consider genes that pass our Bonferroni corrected threshold (at significance level $\alpha = 0.05$ and with a diameter of $D = 34$, our Bonferroni corrected threshold is $log_{10}(p) \geq 2.83$).

We used the DOSE R package [2] to analyze the enrichment of these genes with Disease Ontology (DO) terms [3]. We found that the 379 genes that pass our Bonferroni corrected threshold in at least one bladder cancer were significantly enriched with the DO term "bladder cancer" (DOID:11054) ($p = 1.05 \times 10^{-7}$), supporting the biological relevance of genes identified by GeneSurrounder. Furthermore, our method found three genes, *C2*, *ITGAM* and *VIM*, that pass our Bonferroni corrected threshold in all three studies (Table S2). *C2* plays a role in inflammation and removing debris from cells and tissues. *ITGAM* plays a role in cell adhesion molecules and transcriptional misregulation in cancer. *VIM* plays a role in cell attachment, migration, and signaling and microRNAs in cancer. As we are comparing samples between superficial-vs-invasive bladder cancer (superficial bladder cancer has not grown into the main muscle layer of the bladder, whereas invasive bladder has grown into the main muscle layer), the finding of these three genes from studies of superficial-vs-invasive bladder is sensible and suggests that GeneSurrounder is able to accurately identify mechanistically relevant genes. A table of the full results is provided as an additional file [see Additional file 4, Additional file 5, Additional file 6].

GeneSurrounder results represent a true integration of pathway and expression data

The method that we have developed combines gene expression data with an independent network model. To investigate whether our results are driven solely by either the network or the expression data or represent a true integration of biological knowledge (the pathway networks) and experimental data, we consider the association between our results, the centrality, and the differential expression for each gene. If the results were driven solely by the network, the evidence a gene is a disruptive gene would correlate strongly with its centrality in the network. We therefore calculate the correlation between our results and two different measures of centrality. If the results were driven solely by the expression data, the evidence a gene is a disruptive gene would correlate strongly with its differential expression We therefore calculate the correlation between our results and the differential expression for each of the studies. The results are given in Table S3. We find that for each of the studies, the correlations are small (at most +0.101), confirming that GeneSurrounder is not driven solely by network features or the expression data, but rather represents a true integration of biological knowledge (the pathway networks) with experimental data.

GeneSurrounder findings are more concordant than differential expression analysis

The intuition underlying evaluating cross-study concordance is that methods that detect true biological signals should find them across different data sets measuring the same conditions. To investigate the cross-study concordance of our analysis technique (i.e. its consistency across different data sets measuring the same conditions), we consider each pair of the three studies and calculate the correlation between our results. As a point of reference, we also calculate the correlation between the gene level statistics obtained using the customary t-test for differential expression. The results are given in Table S4. As mentioned earlier, methods that do not take into account systems-level information tend to have poor agreement between studies because the individual genes contributing to disease-associated mechanisms can vary from one study to the next. Indeed, we find that the cross-study concordance of differential expression results is remarkably low (Table S4). By contrast our method is more consistent than differential expression analysis. This cross–study concordance suggests that our method reliably detects biological effects reproducibly across studies.

GeneSurrounder findings are more concordant than LEAN

We also compare GeneSurrounder to LEAN, a recent method that also attempts to integrate gene expression and network data to identify significant genes. In contrast to our method, LEAN considers only the immediate neighborhood (i.e. at a radius of one) and assesses the enrichment of significant genes. To compare the performance of our analysis technique to LEAN, we compare their respective cross-study concordances. To ensure comparability between our method and LEAN, we use the same network and expression data for inputs to LEAN that we used for GeneSurrounder. Again, we consider each pair of the three studies and calculate the correlation between our results and the correlation between results of LEAN [4] (which is available as an R package on CRAN). The results are given in Table S4. We found that GeneSurrounder is more consistent than LEAN. That is, the list of "disruptive" genes detected by GeneSurrounder are more reproducible across studies than both differentially expressed genes and the results from LEAN.

Tables

GEO Accession No.	N(superficial)	N(invasive)
GSE13507	103	62
GSE19915.GPL5186	38	41
GSE32894	213	93

Table S1: **Bladder cancer datasets used in this study**: Comparisons were made between superficial and invasive bladder cancer using public data. Superficial bladder cancer has not grown into the main muscle layer of the bladder and invasive bladder cancer has grown into the main muscle layer of the bladder. Sample sizes for each group in each dataset are given. (GSE19915.GPL5186 originally had 43 superficial samples and 45 invasive samples, but samples with missing data for 25% or more of the genes were filtered out.) The data are publicly accessible and available as part of the curatedBladderData package [1] .

	$-\log_{10} p^{\mathrm{GS}}$		
Gene	GSE13507	GSE32894	GSE19915.GPL5186
C2	2.994	3.864	2.859
ITGAM	2.859	3.452	3.154
VIM	3.314	3.019	3.944

Table S2: **"Disruptive" disease genes in bladder cancer consistently found by GeneSurrounder**: At a threshold of $p = 0.05$ and with a diameter of $D = 34$, the Bonferroni corrected threshold is $-\log_{10}(p) \geq 2.83$. Listed are the genes that pass this threshold in all three studies.

Network/Gene Statistic	GSE13507	GSE31684	GSE19915.GPL5186
Degree Cor.	+0.070	+0.089	+0.045
Betweenness Cor.	+0.038	+0.038	+0.036
p_{DE} Cor.	+0.101	−0.033	+0.096

Table S3: **Correlation between GeneSurrounder results and network/gene statistics**: The three columns are the rank correlation between GeneSurrounder results (p^{GS}) and network/gene statistics (Degree, Betweenness, and p^{DE}) across all genes in each dataset. The Degree and Betweenness are two different network centrality measures. The Degree is the number of connections a node has and the Betweenness is the fraction of shortest paths that passes through the node. p^{DE} is the p-value obtained from a standard differential expression t-test.

Bladder Cancer Study Pair	p^{GS} Cor.	p^{DE} Cor.	p^{LEAN} Cor.
GSE13507 - GSE32894	+0.276	−0.045	+0.023
GSE13507 - GSE19915.GPL5186	+0.206	+0.058	+0.154
GSE32894 - GSE19915.GPL5186	+0.296	+0.016	−0.076

Table S4: **Cross study concordance of GeneSurrounder results compared to differential expression analysis and LEAN**: The columns p^{GS} Cor., p^{DE} Cor., and p^{LEAN} Cor. are the Spearman rank correlations respectively between the results obtained from GeneSurrounder, differential expression analysis, and LEAN for each study pair.

References

[1] Markus Riester, Jennifer M Taylor, Andrew Feifer, Theresa Koppie, Jonathan E Rosenberg, Robert J Downey, Bernard H Bochner, and Franziska Michor. Combination of a novel gene expression signature with a clinical nomogram improves the prediction of survival in high-risk bladder cancer. *Clin. Cancer Res.*, 18(5):1323–1333, March 2012.

[2] Guangchuang Yu, Li-Gen Wang, Guang-Rong Yan, and Qing-Yu He. DOSE: an R/Bioconductor package for disease ontology semantic and enrichment analysis. *Bioinformatics*, 31(4):608–609, February 2015.

[3] Lynn Marie Schriml, Cesar Arze, Suvarna Nadendla, Yu-Wei Wayne Chang, Mark Mazaitis, Victor Felix, Gang Feng, and Warren Alden Kibbe. Disease ontology: a backbone for disease semantic integration. *Nucleic Acids Res.*, 40(Database issue):D940–6, January 2012.

[4] Frederik Gwinner, Gwénola Boulday, Claire Vandiedonck, Minh Arnould, Cécile Cardoso, Iryna Nikolayeva, Oriol Guitart-Pla, Cécile V Denis, Olivier D Christophe, Johann Beghain, Elisabeth Tournier-Lasserve, and Benno Schwikowski. Network-based analysis of omics data: The LEAN method. *Bioinformatics*, 26 October 2016.
